# The Business Department of Our Journal

**Published:** 1858-12

**Authors:** 


					﻿EDITORIAL DEPARTMENT.
The Business Department of our Journal.—Since we assumed the entire
responsibility of this Journal, our editorial duties proper, have been much
augmented by a necessity of transacting some of the business connected with
its publication. Though we have succeeded in regularly issuing it at the
first of every mcnth, and it is a justice which we owe to the enterprising
publishers to say, that it has been issued in a style not surpassed by any of
its class in this country; and though we fervently hope that we have done
our best in its editorial management, yet we would have had a much lighter
task, and perhaps would have performed it more satisfactorily, if we had
been entirely relieved from every thing of a business character. We are
confident, too, that the latter department would have been much more flour-
ishing if it had been taken in charge by a more competent person, as we do
not claim to be a striking exception to the rule, that professional men, and
especially medical men, are not particularly keen in the ordinary transactions
of life.
The house by which this Journal has been published from its commence-
ment, nearly fourteen years ago, and to the energy of which it owed a mea-
sure of the standing it at once assumed among the periodical medical litera-
ture of the land, has long urged upon us the advisability of associating
with ourselves an energetic business man in the capacity of publisher; and
we are now happy to announce that we have made such an arrangement
with one who is no stranger to any of our subscriber, Mr. A. I. Mathews,—
whose advertisement has appeared on the outside of our cover from the very
first number.
By thus adding to our able list of collaborators, who have given our pub-
lication such an enviable literary standing, the services of an active business
man in the financial department, we will be able to present extraordinary
advantages both to subscribers and advertisers; and to subscribers especially,
facilities for obtaining every variety of information, such as are presented
by no other Journal in this country. Our subscription list is already large,
and we will be enabled to make it still larger by presenting the claims of
this Journal to almost every regular physician in the country; by this
we will give advertisers the means of reaching as many physicians by pub-
lishing in our Journal as in any other; and to subscribers then, we will be
able to present the claims of nearly every good mechanical invention, or of
any discovery which is important to the profession; as we will make it for
the interest of all such to advertise with us, and will permit none of a
doubtful character to appear. One half interest in the Journal has been
purchased by Mr. Mathews, who will relieve us of every care, excepting those
purely editorial; thus we will be enabled to give our entire attention to that
department, with no anxiety as regards the other.
To Subscribers, we present the following advantages: A Journal, to the
editorial management of which we are enabled to devote our entire atten-
tion, aided by as able a corps of collaborators as any in the country; having
the advantage of the clinics at two hospitals, and the proceedings of the
Buffalo Medical Association. The establishment of a universal agency for
our subscribers. Mr. Mathews will procure for them every thing which is
required by the practitioner; be will supply any drug or chemical, manu-
factured in this or any other country; any surgical, obstetrical, or dental
instrument; surgical apparatus of all kinds; will procure American, French,
English, or German publications; will subscribe to any American or Foreign
periodical; will procure chemical analyses of every character by Prof. Had-
ley, of the Medical Department of the University of Buffalo; and, in short,
we will present to our subscribers residing in the most remote parts of the
country, the advantages, in the above points, which are enjoyed by a resident
of the city of New York. It will be necessary only to write to the Journal
enclosing two stamps, one for the reply, and one for the communication
which it may be necessary to make with manufacturing or publishing houses.
There will, however, be few articles which he will be unable to supply from
his own stock.
With these advantages, we look forward to an increase in the prosperity
of the Journal, and we pledge for ourselves and for the publisher, on the
one hand, every effort to sustain its reputation in a literary point of view;
and on the other, a prompt attention to every thing connected with its busi-
ness transactions, and the immediate execution of all commissions entrusted
to us by subscribers.
Hereafter, all communications relative to the editorial department, will be
directed as before, to the editor; all remittances, and communications, relat-
ing to the business department, will be addressed to A. I. Mathews, Pub-
lisher. The price of the Journal will remain the same, $2.50 in advance, or
$3.00 at the end of the year. We hope, however, and shall expect, to
receive advance payment in nearly all cases.
Buffalo Medical College. — It will be gratifying to the friends of this
institution to learn that the class for the present session presents a decided
increase from last year. The introductory was delivered on the first Wed-
nesday in November, by Dr. Theophilus Mack, Lecturer on Materia Medica
and Therapeutics, to a good audience, composed of the class and some of our
citizens. It is unnecessary to say that this was an able production, espe-
cially as we have asked aud obtained the privilege of publishing it in our
Journal, when our readers will have an opportunity of passing judgment on
its merits. It will appear in our January number. The following corres-
pondence explains itself:
University of Buffalo, Medical Department,
November 15th, ’858.
Theophilus Mack, M. D.,
Dear Sir,—The class, having heard with great pleasure and benefit your
interesting Introductory Address to the present term, have, at a special
meeting, appointed the following committee for the purpose of procuring a
copy for publication.
In accordance with their instructions, we therefore earnestly request that
you will send us a copy for that purpose.
Yours very respectfully,
George A. McDonell,
Lucien A. Damainville,
Joseph W. Robinson,
L. N. Bates,
Frank Hodge.
St. Catherines, C. W.,
16th November, 1858.
Gentlemen,—I have much pleasure in acquiescing to your request. I
feel highly honored in your considering worthy of publication my modest
attempt at a “ Relief Karte ” of the more salient features of a profession in
the honest exercise of which I hope soon to find every member of your
class.
I have the honor to be,
Gentlemen,
Your obedient servant,
Theoph. Mack.
Messrs. Geo. A. McDonell,
Lucien A. Damainville,
Joseph W. Robinson,
Newton L. Bates, and
Frank Hodge,
Members of Committee of Class, Med. Dep’t University of Buffalo.
New York State Inebriate Asylum.— The corner stone of this institu-
tion was laid on the 24th of September last, with the impressive Masonic
ceremonies. The Grand Master of the State of New York, and many other
high dignitaries of the order, officiated in the exercises. Speeches were
made by the Hon. Edward Everett, Hon. B. F. Butler, Hon. D. S. Dickin-
son, Dr. John W. Francis, and the Rev. Dr. Bellows. A poem was also
read by Alfred B. Street, Esq. These, with the Masonic ceremonies and
speeches from some of the order, made the exercises extremely interesting
and impressive. We learned from one who had the good fortune to be
present, that the speech by Everett was one of his happiest efforts. A
speech from this great man alone, would give interest to such exercises, and
the happy efforts by the remaining orators, combined to make the affair
most satisfactory and successful. The distress, both mental and physical, to
which the vice of intemperance will inevitably lead, is brought more forci-
bly to the mind of no class of men, than to the physician. We know the
terrible diseases to which it gives rise; we see the misery and desolation
which is brought into a virtuous home by the demon of drink, where it is
concealed from all the world beside. The sacred ties of the family physician
lead the unhappy to unburden their heart, to lay bare all their woe, and to
ask him, almost as a superior being, if there be no remedy. A man, and a
man of intellect and education, can see himself the cause of incalculable
unhappiness, can wish and pray to have strength to resist, yet the hold
which a habit of stimulating obtains upon the organism is of such a charac-
ter, that it requires the strongest will to give up a habit, which an abused
constitution reminds him every moment that he has made almost a physical
necessity. The impossibility of legislating successfully against this evil, has
been abundantly proven; we, however, can do something, as has been shown
.by the success in many instances of institutions for inebriates in other coun-
tries. Such a movement on the part of the profession in this country, will
reflect upon them a lasting honor, and we all hope and pray it may be
successful.
Case of Imperforate Rectum. Letter from Dr. Jones, of Charlotte,
Monroe County, N. Y.
Editor of Buffalo Medical Journal.
Dear Sir: I have met with a very remarkable case in my practice, and if
you think it worth publishing, you are at liberty to do so.
On September 18th, 1858, Mrs. Nelson was delivered, after a short labor,
of a fine healthy boy, weighing nine and a-half pounds, plump and fat. The
child seemed quite well for two days, nursed two or three times, slept well, and
was quiet with the exception of occasional turns of vomiting a dark slimy
substance, as infants frequently do at that age. After two days, we thought
it should have its bowels evacuated, and therefore administered a laxative,
which was speedily rejected. Another was given, but also rejected. The
vomiting continued, bringing up a dark offensive substance, resembling me-
conium. We then commenced with enemata, which were not retained a
moment. After having made repeated injections, failing to get any feces, I
introduced a bougie about two and a-half inches, into the rectum, when I
met with an obstruction. After ten days, the parents thought an operation
might be performed to relieve the child, and Dr. Carpenter was called in.
After examining the rectum, we passed a small gum-elastic catheter up the
bowel, as we thought, about eight inches. Dr. C. thought it should remain
for four or five hours. At the expiration of that time I removed it and
found that it had coiled itself in the recium, which was very easily explained
when the instrument became warm and soft. I judged from this, and pre-
vious examinations, that there was a permanent obstruction in the rectum.
After twelve days suffering, the child keeping nothing on its stomach, and
nothing passing its bowels, it died on the morning of the twelfth day.
I obtained the consent of the parents to make a post-mortem examina-
tion, -which was done in the presence of two old ladies. The stomach and
bowels were in a normal state until I came to where the sigmoid flexure of
the c Ion should have been, and here was wanting about three inches of
intestine. The end of the colon was smoothly sealed over, making its termi-
nation in a bag. Over the upper end of the rectum was a strong thick
membrane. The kidneys, ureters, bladder and urethra, were normal. The
bowels were very much distended with gas. I was permitted to make no
further examination.
I am pleased that I was allowed to make the examination, on account of
the gossiping of a certain homoeopathist, who said, “he could have cured
the child, that he had had a hundred such cases.'’ I cannot say that I have
had eleven hundred obstetrical cases, but I have never met with such a case
before. I have read of them and presume they have been met with
frequently.
Yours, &c.,
A. Jones, M. D.
The above is indeed an interesting case. Imperforate rectum is quite a rare
disease, and with the exception of a few cases where the bowel terminates
just beneath the skin, are not amenable to an operation. We believe, indeed,
that most surgeons recommend to leave them alone, and of course death is
the inevitable result. In this case, we conceive that the post-mortem exam-
ination proved that an operation would have been unsuccessful. The alter-
native of an artificial anus is too disgusting to be taken for a moment into
consideration. Conceive of the responsibility of entailing such a loathesome
disease upon a fellow creature I Who is there who would not a thousand
times prefer to have died in infancy? The subject of operation in cases of
imperforate rectum was brought up a short time since, before the Boston
Society for Medical Improvement. We have not the reports at hand, but
think that in most cases the operations were unsuccessful.
In another point of view, the case reported by Dr. Jones, gives us a text.
The meddlesome interference of irregular practitioners, who are always too
ready to play upon the hopes of the friends of patients laboring under a
serious disease. This precious homoeopathist could cure the child, “ he had
cured a hundred such cases.” This is the invariable cry, and often are the
minds of the friends filled with remorse, as well as the standing of the prac-
titioner injured, by such bare-faced effrontery. A post-mortem examination,
however, will bring the truth to light, and it not only defends the reputation
of tlie practitioner, but must be a great relief to friends who otherwise might
not have believed that all had been done.
It is only a few days ago that a patient from whom we had removed the
matrix of the nail of the great toe, for onychia maligna, after the nail had
previously been removed by the ordinary operation, and after she had suf-
fered for three year ', told us that a homoeopathic practitioner volunteered
the valuable information, that now her toe was healed up she would die of
consumption. This professional gentleman was working with her husband
in a blacksmith’s shop, only a short time before, and from thence proceeded
to practice medicine, when he had promised to cure her toe himself with the
inevitable sugar of milk. To be classed with such animals is humiliating,
but it is one of the crosses which we knew we should have to bear when we
took up our profession, ar.d we suppose we have no right to complain.
Meeting of the Medical Society^ of South- Western New York.— Every
half year we receive an account of a meeting of this society, and judge from
what we see that it is always a pleasant affair. When doctors feel a dispo-
sition to come together as brethren, they are the most companionable of
men; good feeling alone prevails, and this appears always to be the case
with our brethren of South-Western New York.
The society met on the first Wednesday in November, and elected the
following gentlemen officers for the ensuing year:
President, H. M. T. Smith, Dunkirk.
Vice-President, Edson Boyd, Ashville.
Secretary and Treasurer, Charles K. Irwin, Dunkirk.
Executive Committee, Dr. G. C. Bennet, Quincy,
“ A. K. Avery, Forestville,
“ G. S. Harrison, Sinclearville.
Com. of Arrangements for 1859, Dr. Boyd, Ashville,
Dr. Thomas, Dewittville,
“ Fenwick, Mayville.
The following resolutions, among others, were unanimously adopted:
Resolved, That the members of the Medical Society of South-Western
New York will not, in any instance, visit, examine or prescribe for a patient
of an irregular practitioner of medicine, until the patient, or his or her guard*
ian, shall have assured us of the discharge of the irregular practitioner, and
our employment.
Resolved, That the Medical Society of South-Western New York endorse,
as professional, the administration of chloroform in obstetrics; and we assure
the public that its use requires the same, and no more skill than that of
other potent remedies; and that we recommend, as safe administrators of it,
any and all regular members of our society and profession as being equally
competent, who desire to assume the responsibility of its administration.
After the meeting, the members, with their wives and invited guests, sat
down to an excellent dinner, which was enlivened by good speeches by the
president, Dr. Smith, and Dr. Washburn, as well as some who were not
members of our profession. We are sorry to be only able to speak in terms
of general commendation of these responses, which are published in full in
the “Dunkirk Press and Western Argus.”
We like the spirit of the first resolution: too often is the practitioner neg-
lectful of inquiring, when called, if any other practitioner, and especially an
irregular, is in attendance. We are thus subjected to the unpleasant chance
of meeting a quack at the house of our patient, and often under the disagree-
able necessity of controling our desire to forcibly eject him.
We do not know what some of our Philadelphia, Boston, or New York
friends would say to the second resolution. It is now becoming quite com-
mon in these cities, to repudiate chloroform and use nothing but ether, espe-
cially in the hospitals and clinics. The horror of the eminent Professor of
Obstetrics in the Jefferson School, when he barely hears of the contemplated
administration of chloroform, could not be greater than if he were asked to
witness a wilful murder; indeed it is a tradition that he was accustomed to
kill sheep with chloroform before the class, to inspire in them a measure of
detestation of the article. This feeling is very common in Philadelphia,
which we look to rather as the fountain head of medicine in this country.
True it is, that a physician or surgeon, if he administers chloroform frequently,
will sometimes see a case, where the escape from death will be very narrow.
We have seen one or two such instances in the practice of eminent surgeons,
and though we much prefer this form of anaesthetic from its pleasant odor,
its rapidity and certainty of action, and do administer it, we are always
rejoiced when the patient is well cut of danger from its effects. The total
arrest of respiration which sometimes occurs in its administration, and which
frequently continues long enough to excite the most lively apprehension, is
a fearful thing for an operator, and an experience which we hope never to
undergo. For all that, however, chloroform is so much more agreeable,
rapid, and certain in its action than ether, and the cases of death from its
administration, with proper precaution, are so rare, that we always give it
the preference. We regard ether, however, as absolutely without danger.
What the anaesthetic acetone, which is described in Brown-Sequard’s Jour-
nal and which we referred to in our last number, may prove to be, we do
not know; we hope that some agent may be discovered which will prove as
effectual as chloroform, and as safe as ether.
The society adjourned, Nov. 4tb, to meet at Mayville on the second Wed-
nesday in May, 1859.
Letter detailing a case of Criminal Abortion and Infanticide.—We
publish the following letter, which appears to have been called out by our
article on Criminal Abortions, which details one of the most horrible in-
stances of depravity, is rather insanity, which we have ever known. This
is one of the effects of advertising abortion pills; and that such a thing
should ever occur in a civilized land would be a matter of astonishment to
any but the physician, who sees so much misery and crime. Cannot this
matter be remedied, and will not our brother editors aid us in the effort to
awaken public sentiment in this direction?
Dr. Flint—Dear Sir: I see by the two last numbers of the Buffalo
Medical Journal that you have undertaken to correct and change the public
mind, and so do away with a great moral evil (criminal abortions). I am
glad, for, one that the medical faculty are getting aroused to this great
subject, and that they may show its true character and influence, and cor-
rect, if possible, the error. It is a great evil, and there must be measures
and means advanced to check it, or where will it end ?
I will give you the history of a case that occurred to me in my practice
only a year ago.
Mrs. W., aet forty, widow woman, with three children, applied at my office
for medical aid; said she had been unfortunate, and got caught in a bad
scrape, and wished something to produce the desired effect. She was told
that it was very dangerous to give such medicine, and that they were very
uncertain in their effects, as no specific medicine was known that would
produce this effect without danger. She left the office, and nothing more
was heard from her for about two weeks, when I was summoned to her
house as soon as I could get there. I was some twelve hours before I ar-
rived, and when I did, I learned a tale that makes my blood almost chill
when I think of it. She had procured some drug, the character of which
she refused to tell; had taken it and it had the desired effect, i. e., to pro-
duce abortion. She told me that she was alone with her children, who
were asleep. She took the medicine, and the child was born ; that she
knew not what to do with it, and for fear that it would cry and awaken
the other children she muffled its mouth, got off her bed, walked to the
stove, in which there was a brisk fire, removed the griddle or cover, and
placed the child upon the burning coals, and there it remained until it
was burned to ashes. She said it writhed and tried to cry, and she had to
turn away!
To what extent of crime will this debasing practice lead our mothers
and our daughters if not stopped? The foetus was a seven and a half
month. The effect of the medicine which she took produced her death
some hours after. This was her confession.
I could relate other instances that have occurred in my neighborhood, but
they are no more than have occurred to you and to every other medical
man.
I will state what I know of some of the “Royal French Physicians"
who advertise in almost every paper that we pick up, a specific remedy for
suppression of the menses, &c. You doubtless have seen an advertisement
of Dr. Charles S. Gourander, French Physician, Surgeon and Accoucher,
of the celebrated school of Paris, &c. Also, J. B. Bartonie, with an adver-
tisement of like character.
These two eminent physieians are only one person assuming these two
names. I am personally acquaintad with this eminent humbug and do
know that he never read a medical book in his life, neves attended a medi-
cal school, never heard a medical lecture, and yet he advertised as being a
graduate of the Royal College of Paris, (what students she must turn out.)
I have seen this man go to the post-office and get from three to fifteen let-
ters at a time directed to him at N. Y., and forwarded to R., and from each
of these letters he received one dollar with directions to forward to such and
such places his pills with full directions for use.
The most of these letters were from unmarried ladies—at least they were
signed Miss So and So. These letters I have both seen and read. The
pills he used were Wright’s Indian Vegetable. He bought them by the
gross, tore off the wrappers and put on his, and forwarded them to differ-
ent parts of the United States, with directions for use. This will show the
imposition practiced upon the people; but I think that his pills may be
safer and less hazardous to life than the most of preparations used for this
purpose, and by just such quacks as he.
Yours, &c.,	G. H. C.
The North American Medical Reporter. Edited by W. Elmer, M. D.
This is the title of a new journal published by W. A. Townsend & Co.,
New York, the publishers of Braithwaite’s Retrospect. It is to be regretted
that a journal which is evidently designed to be issued in a kind of con-
nection with such a work as Braithwaite’s should not take a more dignified
position than the one before us. It is designed to present an index of the
interesting articles which appear in the various periodicals to which it has
access; so far it is good; but in addition, even the first number, which it
would be policy to keep free from every thing objectionable, we regret to
say, contains many things which the regular profession cannot but condemn.
We should not have mentioned this publication, had it appeared under the
guise of an eclectic or homeopathic journal, but the position which it as-
pires to assume, the circulation which it hopes to obtain among the regular
profession, and especially its apparent connection with the reprint of Braith-
waite’s Retrospect, make it necessary for us to speak of its claims on the
profession. A journal which contains a favorable notice of “The American
Family Physician or Domestic Guide to Health,” by John King, M. D.,
Professor of Obstetrics and Diseases of Women and Children in the
Eclectic College of Medicine, Cincinnati; and the “ Pronouncing Medical
Lexicon,” by C. II. Cleveland, M. D., Professor in the same institution,
can not demand our support or sanction. We say nothing of an editorial
article which reads as though it had been copied from the advertising
sheet of a daily paper, announcing a new hair invigorator, the formula
“ to be found in a subsequent part of this number, which constitutes one of
the best if not the only rational preparation for restoring the hair to its
original or healthful condition.” These are undignified and professional,
and we cannot commend a publication which, like this, either panders to
the interests of an eclectic, or the discoverer of a hair wash, or puts himself
on a level with them, and indorses their opinions.
The Physician's Diary for 1859. Buffalo: Phinney & Co., publish-
ers, No. 188 Main street. 1859.—We have just received this admirable
Physician’s Diary for 1859, and hasten to notice its appearance. This
has been the companion of most if not all our physicians for the past
year or more, and we, for one, can testify to its usefulness and convenience.
It is of a size sufficiently small to be carried in the pocket without in-
convenience, and yet is so admirably arranged as to contain all the notes
which we wish to make in such a form, It is so well known to our-
readers that it is almost unnecessary to recite what it contains. Every phy-
sician should commence the year with one. They may be obtained of dif-
ferent sizes, from Phinney & Co., of this city. The contents are, An Alma-
nac, List of Poisons and their Antidotes, Visiting List and Medical Record,
Obstetrical Record, Nurses’ Addresses, List of Things Lent, and Bills to
be Presented.
To Exchanges and Subscribers.—We are very short of the August num-
ber for 1858, of our Journal, it having been nearly all sent off by mistake,
and should be glad to credit fifty cents on the subscription of any person
who does not bind the Journal and will mail this No. to us. $2 and the
August number for 1858, will be received for subscription for the next vol-
ume. If any of our exchanges can spare us this number, of which every
one counts with us, and will send it to us, it will do us a great service.
Congenital Fissure of the Sternum.—We have received a pamphlet con-
taining extracts from the Album of E. A. Groux, who has a congenital fis-
sure of the sternum, and suppose that the profession of this city will soon
have an opportunity of examining this remarkable case. The extracts are
interesting observations on his case by some of the most distinguished Ger-
man, Spanish, French and English physicians, and indicate the great inter-
est which Mr. Groux has excited among members of the profession wherever
he has exhibited himself.
				

